# Alternative splicing and protein function

**DOI:** 10.1186/1471-2105-6-266

**Published:** 2005-11-07

**Authors:** AD Neverov, II Artamonova, RN Nurtdinov, D Frishman, MS Gelfand, AA Mironov

**Affiliations:** 1 State Scientific Center GosNIIGenetika, 1st Dorozhny proezd 1, Moscow, 117545, Russia; 2 Institute for Bioinformatics/MIPS, GSF – National Research Center for Environment and Health, Ingolstädter Landstraße 1, 85764 Neuherberg, Germany; 3 Department of Bioengineering and Bioinformatics, M.V.Lomonosov Moscow State University, Vorobievy Gory 1–73, Moscow, 119992, Russia; 4 Department of Genome Oriented Bioinformatics, Technical University of Munich, Wissenschaftszentrum Weihenstephan, 85350 Freising, Germany; 5 Institute for Information Transmission Problems RAS, Bolshoi Karetny pereulok 19, Moscow, 127994, Russia

## Abstract

**Background:**

Alternative splicing is a major mechanism of generating protein diversity in higher eukaryotes. Although at least half, and probably more, of mammalian genes are alternatively spliced, it was not clear, whether the frequency of alternative splicing is the same in different functional categories. The problem is obscured by uneven coverage of genes by ESTs and a large number of artifacts in the EST data.

**Results:**

We have developed a method that generates possible mRNA isoforms for human genes contained in the EDAS database, taking into account the effects of nonsense-mediated decay and translation initiation rules, and a procedure for offsetting the effects of uneven EST coverage. Then we computed the number of mRNA isoforms for genes from different functional categories. Genes encoding ribosomal proteins and genes in the category "Small GTPase-mediated signal transduction" tend to have fewer isoforms than the average, whereas the genes in the category "DNA replication and chromosome cycle" have more isoforms than the average. Genes encoding proteins involved in protein-protein interactions tend to be alternatively spliced more often than genes encoding non-interacting proteins, although there is no significant difference in the number of isoforms of alternatively spliced genes.

**Conclusion:**

Filtering for functional isoforms satisfying biological constraints and accountung for uneven EST coverage allowed us to describe differences in alternative splicing of genes from different functional categories. The observations seem to be consistent with expectations based on current biological knowledge: less isoforms for ribosomal and signal transduction proteins, and more alternative splicing of interacting and cell cycle proteins.

## Background

The current estimates of the prevalence of alternative splicing in the human genome fall into the interval 35–60% [1-7], whereas the estimated number of human protein-coding genes has decreased from more than 100 thousand [8] through 30–35 thousand [3,9,10] to 20–25 thousand [11,12]. Thus alternative splicing emerges as a major mechanism of generating protein diversity. Continuing sequencing of ESTs, whose number currently approaches 4 million, uncovers rare, tissue- and stage-specific isoforms. On the other hand, a considerable number of ESTs seem to arise from experimental artifacts (genome contamination, unspliced transcripts, computational errors leading to mis-alignment and clustering ESTs from paralogous genes, etc.) or errors of the cellular splicing machinery itself (so-called aberrant splicing). The latter might be a relatively frequent event, as there exists a special mechanism for surveillance of splicing errors, leading to elimination of aberrant mRNA isoforms by nonsense-mediated decay [13].

The algorithms for construction and enumeration of full-length isoforms should take into account as many sources of errors as possible. In early studies EST contigs were constructed as consensus exon sequences so that each exon was used only once. This precluded combinatorial explosion, but led to generation of short single-exon contigs, and besides did not allow for enumeration of isoforms. The use of contigs for estimation of the number of isoforms [14] leads to estimates that depend on EST coverage [15]. Recent algorithms construct splicing graph whose vertices correspond to sites and edges to sequence fragments in such a way that each path in this graph corresponds to a possible isoform [16-18]. Here we apply the IsoformCounter algorithm that constructs the splicing graph aligned to the genomic sequence and computes the number of possible protein isoforms. The latter procedure employs coverage-dependent thresholds for filtering artifacts.

We used IsoformCounter to compute the number of isoforms of alternatively spliced genes from the EDAS database [19] for different functional classes of proteins from GO [20]. We estimated the probability of spliceosome error (1.2%) and suggested a simple probabilistic model for filtering exons and introns obtained by EST-genome spliced alignment. As a result we have obtained a robust method for enumeration of protein isoforms independent of EST coverage. We observed that the fraction of genes with less alternative splicing (one or two protein isoforms per gene) is higher in "Small GTPase-mediated signal transduction" and "Ribosome" classes, and lower in the "DNA replication and chromosome cycle" class, compared to the average distribution. We also analyzed the correlation between alternative splicing and protein-protein interactions and demonstrated that interacting proteins are more likely to be encoded by alternatively spliced genes.

## Results

### Algorithm for counting alternatively spliced isoforms

The following terms will be used. *Exons *and *introns *are genome fragments that correspond to exons and introns respectively in spliced alignment of some EST, mRNA, or protein with genomic DNA. *Initial *and *terminal exons *correspond respectively to first and last exons in spliced alignment. *Support *of an exon is the number of clone libraries containing ESTs whose spliced alignments contain exactly this exon; specific cases are spliced alignments with mRNA and proteins that generate exons of *mRNA *and *protein support *respectively. Protein-supported exons are ascribed the reading frame derived from protein spliced alignment, whereas EST- and mRNA-supported exons are considered as triples with the same splicing sites and all possible reading frames.

For each gene (genomic fragment and corresponding ESTs, mRNAs and proteins) the algorithm constructs the *splicing graph*. Each splicing site corresponds to three vertices of this graph (for three possible positions relative to the reading frame), and its edges are exons and introns. The reading frames of vertices and corresponding edges are consistent. Thus each path through this graph corresponds to a candidate mRNA isoform. There is also a special type of vertices, start and stop codons, that open and close a reading frame respectively. A *protein isoform *is a path starting at a start codon or 5'-boundary of an initial exon and ending at a stop codon, with an additional condition that initial and terminal exons are supported by at least two clone libraries.

The IsoformCounter algorithm filters isoforms (paths) unlikely to be expressed as a functional protein. The filters are listed below.

#### (1) Start codons (Fig. 1)

**Figure 1:**
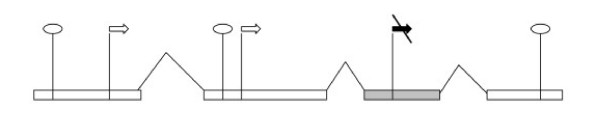
**Representation of start and stop codons in the splicing graph**. Rectangles: exons. Angle lines: introns. Circles: stop codons. Arrows: start codons. Filled arows: start codons generated by alignment with proteins. Crossed arrows: start codons in-frame with an upstream acceptor site or a start codon. The following situations are considered and marked on the scheme:. (1) In each exon, for each possible reading frame, a stop codon closing this reading frame generates a *stop-vertex*. (2) If an exon contains a start codon preceded by a stop codon in the same reading frame, a *start-vertex *is generated. If a start codon coincides with the beginning of a protein-genome alignment, it generates a *start-vertex *irrespective of upstream stop codons (gray exon). In the latter case, no additional filters are applied (see the text). (3) Any start-vertex that is in-frame with an upstream acceptor site or a start codon is removed (crossed arrow).

An ATG codon generates a vertex only if it is confirmed by spliced alignment with a protein or if it is preceded in an exon by a stop codon in the same reading frame. Thus a protein isoform starts with the leftmost methionine in a given reading frame or by a methionine supported by a protein spliced alignment. To account for a possibility of insufficient coverage of a gene 5'-region, we also allow a protein isoform to start at a 5'-end of an initial exon, if this isoform does not contain in-frame protein-supported methionine codon.

#### (2) Initiation of translation (Fig. 2)

**Figure 2:**
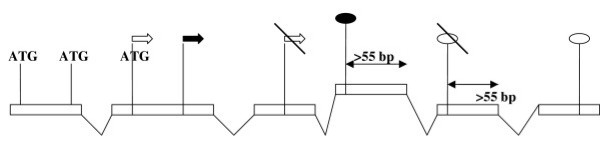
**Filters on translation initiation for start-vertices and nonsense-mediated decay for stop-vertices**. Circles: stop-vertices. Arrows: start-vertices. (1) Filters are not applied to vertices supported by protein-genome alignment (filled symbols). (2) Translation initiation filter: start-vertices preceded by at least two ATG codons in at least one path are removed. (3) Nonsense-mediated decay filter: stop-vertices for which the distance to the nearest donor site exceeds 55 nucleotides are removed.

Translation initiation of most eukaryotic mRNAs uses the so-called linear scanning mechanism: 40S ribosomal subunit binds the cap structure at the 5'-terminus of the mRNA and slides to the proximal ATG codon, where it initiates translation, if the codon is in a favorable context [25]. Stable hairpins and upstream ATG codons decrease the efficiency of the linear scanning. A minor fraction of mRNAs (2–8%) contain internal sites of translation initiation [26,27]. To emulate this mechanism, IsofomCounter considers only isoforms with at most two upstream ATGs, unless the start codon is supported by a protein.

#### (3) Short protein isoforms

We assume that alternative splicing may eliminate up to 50% of the *average protein length*, that is, the average length of proteins from RefSeq corresponding to the given gene. If the gene does not have RefSeq entries, the average length of corresponding proteins in EDAS is used. Further, no proteins shorter than 33 amino acids were considered.

#### (4) Consistency with proteins

We required that an isoform had at least one amino acid overlapping with a known protein encoded by the gene. This filter is sufficient to remove long open reading frames in 5'-untranslated regions. On the other hand, selecting a stricter threshold would lead to the loss of some known isoforms. Further, we require that there are no conflicts between reading frames generated by intersections with proteins.

#### (5) Premature termination of translation (Fig. 2)

It is known that transcripts containing premature stop-codons due, in particular, to spliceosomal errors, are degraded by a specific mechanism of nonsense-mediated decay (NMD) [13]. As we have no criterion for distinguishing between functional and aberrant alternative splicing, we implemented a filter imitating the NMD action, requiring that the last exon-exon junction in an isoform were at most 55 nucleotides downstream its stop-codon. As above, this filter was not applied to protein-supported isoforms.

#### Counting protein isoforms

To compute the number of protein isoforms, IsoformCounter finds the number of all paths in the splicing graph and subtracts the number of paths not consistent with any known protein, see filter (4). The former value is computed in linear time (respective the number of edges) by dynamic programming. To compute the latter value, IsoformCounter constructs a subset *A *of acceptor sites of protein-supported exons and exons that overlap the former in consistent reading frame. Then the complement set A* of acceptor sites is determined, and the number of paths coming through A* (and not through any site from *A*) is calculated by dynamic programming. By definition, these paths are not consistent with any known protein.

#### Computing the numer of alternative regions in the longest protein isoform

Constitutive regions are exon fragments whose genome projections never overlap with introns or intergenic spacers. The procedures described above allow for segmentation of the longest protein isoform into constitutive and alternative regions so that only valid isoforms that have passed all filters are taken into account.

#### Normalization procedure for EST-derived exons and introns

The following model was considered. Let α be the probability of splicing error (loss of a site), and let ξ be the expression level (average number of gene transcripts per cell). We assume that ξ = *f*(*N*), where *N *is the observed number of ESTs. Let *P*(*N*) be the probability that the cell contains at least one aberrantly spliced transcript, *P *= 1 - (1 - α)^ξ^. Then the probability that the error occurred in an interval supported by *k *clone libraries is P^*k *^≤ *β*, where β denotes the significance level, that is, the probability with which we accept a splicing error. Solving this inequality with respect to *k*, we obtain *k*(ξ) ≥ ln(β) / ln(1 - (1 - α)^ξ^). We estimate the expression level as ξ = *N/5*. To estimate the probability of a splicing error, we considered losses of one or two sites corresponding to a protein-supported intron. The number of such events *N*_*overlap *_can be estimated as the number of ESTs which overlap the intron sites and are not spliced at these sites. This is an overestimate, as real alternative splicing events also are counted. Further, let *N*_*splice *_be the number of ESTs whose spliced alignment contain protein-supported introns. The probability of the spliceosome error was estimated as α = *N*_*overlap*_/(*N*_*overlap*_+*N*_*splice*_) computed as 0.012. Note that one EST could be counted both for *N*_*overlap *_and *N*_*splice*_, so the above value could be an underestimate. Finally, we used the following threshold on the number of clone libraries required to accept an exon (dependent on the EST coverage of the gene): *k*(* N*) = [- 1/ln(1-0,988^*N*/5^)]. Exons and introns confirmed by alignment with mRNA or protein are always accepted.

#### The restricted set of protein isoforms

For each gene in EDAS we constructed the restricted set of protein isoforms that are amino acid sequences are available at [37]. Define *isoform support *as the minimum *support *of *exons *and *introns *forming the isoform. For each edge in the splicing graph (exon or intron) we construct the longest isoform passing through the edge, whose support is not less than the edge support. At each level of support, the restricted set consists of such longest isoforms for all edges of the given support.

### Analysis of alternative splicing in functional categories of genes

When IsoformCounter was applied to all genes from EDAS, in 431 cases (4%) no isoforms were found. This could happen in one of there cases. (1) Protein-DNA spliced alignment does not end at stop codon, and there is no downstream terminal exon where the induced reading frame contains a stop codon. (2) Protein-DNA spliced alignment does not start at methionine, and there is no upstream exon where the induced open reading frame contains an ATG codon preceded by a stop codon (see filter 1 in Methods). (3) Protein-DNA spliced alignment does not start at methionine and the candidate ATG found by the algorithm is eliminated by the filter on translation initiation (filter 2), or the alignment does not end at a stop codon, and the candidate stop codon is eliminated by the filter on premature termination of translation (nonsense-mediated decay, filter 5). The first and second cases are due to incomplete proteins where no candidate start or stop codon could be assigned. In the third case a start or stop codon could be assigned based on spliced alignment with incomplete protein and ESTs, but the obtained reading frame was eliminated by the filters. It may happen if the gene has an internal translation initiation site or a special mechanism to keep the isoform from the NMD degradation. In both cases algorithm needs an alignment with a complete protein. All these genes were ignored.

**Figure 3:**
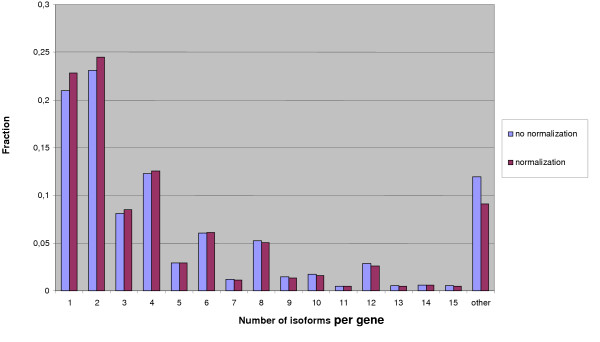
Blue columns: raw data. Red columns: normalized data (see Results). The difference between histograms before and after normalization is weak, because the fraction of highly expressed genes (>400 ESTs) is small (approximately 4%).

**Figure 4:**
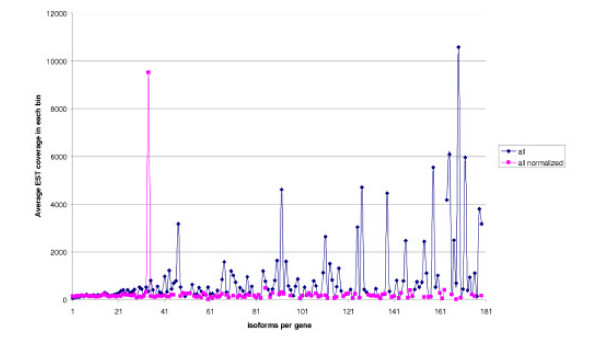
**Correlation between the isoform number and EST coverage**. Blue: raw data (all ESTs). Red: normalized data (coverage-dependent filter on the number of clone libraries supporting exons, see Results). Each dot represents the average EST coverage for genes with the given number of isoforms. The peak in the normalized plot corresponds to the gene "eukaryotic translation elongation factor 1 alpha 1", represented by 18841 EST.

**Figure 5:**
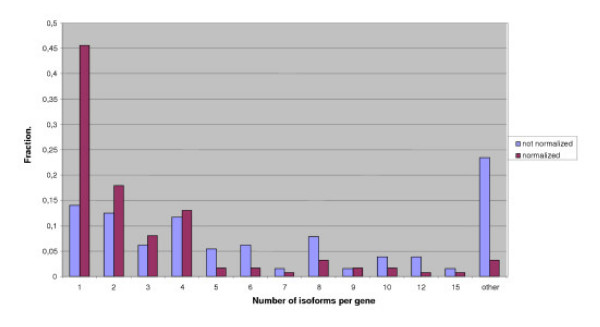
Influence of normalization on the isoform number of proteins from the "Ribosome" GO category. Color code as in Fig. 1.

The obtained distribution of the isoform numbers is shown in Fig. 3 (blue columns). As it is known that the predicted number of isoforms may depend on the EST coverage, we analyzed the dependence between the EST coverage of a gene and the number of isoforms (Fig. 4). The blue plot, corresponding to the initial (raw) data demonstrates that genes with high EST coverage (>900 ESTs per gene) have a large number of isoforms (fall in the tail of the distribution). In particular, this tail contains many genes of ribosomal proteins (Fig. 5), which seems to be an artifact. Indeed, it is highly likely that the high expression level of a gene leads to appearance of relatively rare aberrant isoforms that are not seen for weakly expressed genes.

Thus we believe that the threshold level of EST support for exons and introns should depend on the EST coverage of a gene, so that weakly and highly expressed genes could be comparable. Red columns in Fig. 3 show the number of isoforms with this more stringent threshold. The distributions with raw and normalized data do not differ much, but comparison of two plots in Fig. 4 shows that normalization removes the dependence between EST coverage and isoform number. One functional category strongly affected by the normalization procedure is "ribosome". Further we consider results obtained after normalization.

Most genes (91%) have a relatively small number of isoforms (1 through 15). The number of genes with an even number of isoforms is higher than the number of genes with odd number of isoforms. Indeed, the algorithm assumes independence of individual elementary alternatives, and thus the number of paths between two alternatives is roughly the product of the number of variants. Moreover, most local alternatives preserve the reading frame. Thus, to have an even number of alternatives, it is sufficient to have a frame-preserving local alternative with two variants (e.g. a short cassette exon). 23% of genes had only one functional isoform.

**Figure 6:**
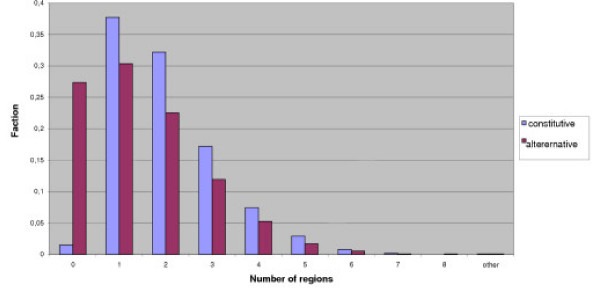
**Number of constitutive (blue) and alternative (red) regions in the longest isoform**. The fraction of completely alternative genes is ~1%.

Fig. 5 shows the distribution of the number of alternative and constitutive regions in the longest isoform. The fraction of genes not containing constitutive fragments at all is ~1%, which shows that the applied filters remove a considerable fraction of aberrant events. Without these filters most genes would contain only alternative regions. The fraction of constitutive genes represented in Fig. 6 is 24%, which is higher than the above estimate. It is caused by the fact that introns not overlapping with the longest isoform are not taken into account. The average number of all (alternative and constitutive) fragments per gene is 3.7.

**Figure 7:**
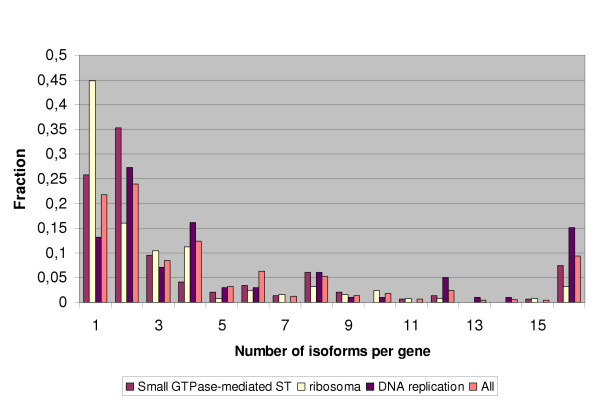
Distribution of the isoform number in GO functional clusters.

We considered the link between protein function and the isoform number. The following functional categories from GO were considered: "Small GTPase-mediated signal transduction" (145 genes), "Catabolism" (512 genes), "DNA replication and chromosome cycle" (99 genes), "Ribosome" (123 genes). Significant differences from the distribution for all genes (p = 0.003 according to the Mann-Whitney U test) were observed for "Ribosome" and "Small GTPase-mediated signal transduction" categories. Both of them contain fewer than expected genes with a large number of isoforms. In particular, there are 46% constititutive genes in "Ribosome", although to very high EST coverage this is observable only after normalization (Fig. 5). Genes from the "DNA replication and chromosome cycle" have more isoforms that the average (p = 0.07 according to the Mann-Whitney U test). In particular, there is a higher fraction of genes with two or more isoforms. The distribution of the isoform number for genes from these categories is shown in Fig. 7.

Of 452 human interaction pairs in the MPPI database, 332 pairs were heterogeneous and different (excluding protein contacts with itself and pairs that differ only by the protein order). LocusLink information was available for 312 of these pairs, containing 386 proteins. Of these proteins, 262 are encoded by genes from EDAS, and pairs with both members present in EDAS form 198 interacting pairs.

No correlation was observed between the number of contacts for a given protein and the number of isoforms or alternative regions (data not shown). However, the probability to be alternatively spliced was higher for genes encoding proteins participating in at least one protein-protein interaction (Table 1). This observation was significant for alternative splicing with all considered levels of support (only proteins, proteins and mRNA, ESTs from different number of clone libraries, ESTs with normalized threshold) at the level <0.1–1% (the highest χ^2 ^= 11.2 for protein-supported alternatives, χ^2 ^from 6.5 through 7.8 for EST-supported alternatives), although the difference between observed and expected numbers is not large (17–30% deficit of constitutive and 10–25% excess of alternatively spliced genes among those encoding interacting proteins). No significant correlations were observed for larger, but noisier non-curated protein-protein interaction datasets DIP and OPHID (data not shown).

**Table 1:** Correlation between alternative splicing (AS) and protein-protein interactions (PPI). Expected numbers under independency assumption are given in parentheses. "EST-N" denotes isoforms with each exon supported by ESTs from at least N clone libraries.

protein data, χ^2 ^= 11.17	No PPI	At least one PPI	TOTAL
No AS	5434 (5408)	122 (= 83% of 148)	5556
At least two AS-isoforms	3692 (3718)	127 (= 125% of 101)	3819
TOTAL	9126	249	9375
			

mRNA data, χ^2 ^= 9.05	No PPI	At least one PPI	TOTAL

No AS	4879 (4856)	107 (= 82% of 130)	4986
At least two AS-isoforms	4360 (4383)	141 (= 120% of 118)	4501
TOTAL	9239	248	9487
			

EST-5 data, χ^2 ^= 6.57	No PPI	At least one PPI	TOTAL

No AS	4463 (4443)	99 (= 83% of 119)	4562
At least two AS-isoforms	4804 (4324)	149 (= 115% of 129)	4953
TOTAL	9267	248	9515
			

EST-3 data, χ^2 ^= 7.79	No PPI	At least one PPI	TOTAL

No AS	4001 (3979)	85 (= 80% of 107)	4086
At least two AS-isoforms	5302 (5324)	164 (= 115% of 142)	5466
TOTAL	9303	249	9552
			

EST-2 data, χ^2 ^= 7.57	No PPI	At least one PPI	TOTAL

No AS	3299 (3278)	68 (= 77% of 89)	3367
At least two AS-isoforms	6063 (6084)	185 (= 113% of 164)	6248
TOTAL	9362	253	9615
			

normalized data, χ^2 ^= 7.23	No PPI	At least one PPI	TOTAL

No AS	2275 (2257)	42 (= 70% of 60)	2317
At least two AS-isoforms	7066 (7084)	206 (= 110% of 188)	7272
TOTAL	9341	248	9589

## Discussion and conclusion

IsoformCounter is a system of filters aiming at distinguishing functional isoforms from non-functional ones. Unlike other programs for isoform generation [18,28] it assumes independence of variants selected at elementary alternatives.

The prevalence of genes with a relatively small number of isoforms agrees with the observation of [29], where all genes had less than 18 isoforms. On the other hand, the prevalence of genes with the even number of isoforms not observed in [29], where the minimal number of isoforms required to explain all local alternatives was computed, as opposed to all isoforms. The fact that 73–77% genes had more than one isoform also is consistent with previous estimates: the fraction of single-exon human genes is 20% [30], and, as the latter are not covered by EDAS, the fraction of alternatively spliced genes is approximately 60% (75% of 80%) [14,31].

It has been reported that alternative splicing tends to affect genes involved in signal transduction [31,32], although no estimates on the significance of these findings was done. We have also expected that there would be considerable differences between such GO categories as "Metabolism" and "Signal transduction". However, this was not observed, probably because these categories are too large and contain many genes with diverse functions and properties. Still, we observed significantly lower number of isoforms for genes from the "Small GTPase-mediated signal transduction" (compared to the distribution for all genes)

The correlation between alternative splicing of genes and protein-protein interactions of encoded proteins was somewhat unexpected, especially given previously described lack of correlation between alternative and contacting regions [33]. Further, although it has been reported that alternative splicing often targets domains involved in protein-protein interactions [34,35], there was no increase in the rate of alternative splicing of such domains.

The effect observed here was not particularly strong (~20–25%), but still statistically significant, and was independent from the support level of alternatively spliced isoforms. On the other hand, the result crucially depended on the reliability of protein-protein interaction data: no correlation was observed for non-curated, large-scale experimental (DIP) or inferred (OPHID) data.

## Methods

The data about alternative splicing of human genes were taken from the EDAS database [19,36]. EDAS contains information about 9986 human genes (9914 with LocusLink identifiers) of which 8324 (83%) show at least some evidence of alternative splicing. The criteria for inclusion of a gene into EDAS were as follows: at least one linked protein sequence, at least one intron in the coding region, and at least 25 ESTs.

The data about protein-protein interactions (PPI) were taken from the manually curated MPPI database [21,39] containing 452 pairs of interacting human proteins. We also considered two PPI datasets, non-curated database of PPI interactions from large-scale experiments (DIP) [22] and predicted PPI (OPHID) [23]. Functional categories of genes were taken from GeneOnthology [20,40].

Spliced alignments were constructed by Pro-Frame [24] (protein-DNA) and Pro-EST [1] (mRNA-DNA and EST-DNA).

## Availability

The software (IsoformCounter) for generating of alternative mRNA isoforms is available for download at [38].

## Authors' contributions

AAM, DF and MSG conceived the project. RNN developed the EDAS database. AAM and ADN developed the algorithm for counting isoforms. ADN analyzed the correlation with functional categories. IIA and DF analyzed the correlation with protein-protein interactions. ADN and MSG wrote the draft. All authors edited the final text.
